# Impact of enterprise digitalization on green innovation performance under the perspective of production and operation

**DOI:** 10.3389/fpubh.2022.971971

**Published:** 2022-11-17

**Authors:** Hailin Li, Hongqin Tang, Wenhao Zhou, Xiaoji Wan

**Affiliations:** ^1^College of Business Administration, Huaqiao University, Quanzhou, China; ^2^Research Center for Applied Statistics and Big Data, Huaqiao University, Xiamen, China

**Keywords:** enterprise digitization, green innovation performance, production and operation, cluster analysis, decision rules

## Abstract

**Introduction:**

How enterprises should practice digitalization transformation to effectively improve green innovation performance is related to the sustainable development of enterprises and the economy, which is an important issue that needs to be clarified.

**Methods:**

This research uses the perspective of production and operation to deconstruct the digitalization of industrial listed enterprises from 2016 to 2020 into six features. A variety of machine learning methods are used, including DBSCAN, CART and other algorithms, to specifically explore the complex impact of enterprise digitalization feature configuration on green innovation performance.

**Conclusions:**

(1) The more advanced digitalization transformation the enterprises have, the more possibly the high green innovation performance can be achieved. (2) Digitalization innovation is the digitalization element with the strongest influence ability on green innovation performance. (3) As the advancement of digitalization transformation, enterprises should also focus on digitalization innovation input and digitalization operation output, otherwise they should pay attention to digitalization management and digitalization operation output.

**Discussion:**

The conclusions of this research will help enterprises understand their digitalization competitiveness and how to practice digitalization transformation to enhance green innovation performance, and also help the government to formulate policies to promote the development of green innovation in the digital economy era.

## 1. Introduction

Since the reform and opening up, the extensive growth model helps China's economy dramatically transform into one of the relatively important economies, but this development model also causes ecological problems like greenhouse gas emissions and excessive resource consumption (1), thus endangering public health and sustainable development. In September 2020, China announced to the world at the general debate of the UN General Assembly that it will strive to achieve carbon peaking by 2030 and carbon neutrality by 2060. In the same year, the Chinese government once again emphasized the need to accelerate the promotion of green and low-carbon development and promote the comprehensive green transformation of economic and social development. In recent years, the COVID-19 epidemic has made people more aware that societal development is always constrained by the natural environment, and economic growth should be coordinated with the protection of the natural ecological environment (2). As the micro-subject of pollution emissions and the micro-engine of economic development, industrial enterprises are obliged to actively practice the concept of green development in production and operation in the face of these green policies, regulations, and issues of concern to the people. Moreover, the innovation activities of these enterprises should also incorporate responses to these policies and take into account social responsibilities including protecting the environment, thereby transforming them into green innovations. Green innovation is not only an effective way to improve organizational performance (3), but also an important strategic catalyst for sustainable development (4), which becomes the focus of academic research and corporate practice (5).

In addition, with the emergence and development of various digitalization technologies such as AI, cloud computing, and Internet of Things, many countries have also paid attention to them and promoted various related policies. For instance, Industry 4.0 proposed by Germany in 2013 emphasized the development of modern industry by intelligence and digitalization. The Chinese government's proposal for Intelligent Manufacturing 2025 also emphasizes the introduction of digitalization technologies into enterprises in the real economy. At the economic level, the importance of the digital economy has also increased. Further taking China as an example, the scale of the digital economy will reach 19.2 trillion yuan in 2021, accounting for 38.6% of the GDP. These external policies and economic environment will inevitably affect the production and operation of enterprises. Existing research has shown that with the understanding and application of digitalization technology, the business model, production operation, and organizational practices of enterprises have undergone major changes (6), which also help them to obtain greater competitive advantages (7). Moreover, innovation activities of enterprises are gradually dominated by digitalization (8), so the complex impact of digitalization transformation on enterprise innovation has certain research value and is worthy of discussion by researchers (9). As green innovation is a part of enterprise innovation activities, whether digitalization has an impact on it also needs to be discussed.

All in all, in the context of digital economy and green innovation becoming more popular, digitalization and green innovation will have an important role on enterprise development and social progress, and the relationship between them needs to be fully clarified. Specifically, how companies should choose an appropriate digitalization transformation path to help improve green innovation performance is worthy of scholars' discussion. This is directly related to the healthy development of enterprises and the sustainable development of the economy, and may even be related to the public health. Therefore, our research aims to address this issue and analyze the hidden impact of digitalization on green innovation, thereby providing theoretical contributions to academia and recommendations for corporate practice.

In order to successfully complete the objective of this research and determine the parts of the research, the background is worth reviewing again. In the era of information explosion, the investigation of the research subjects is carried out in a multi-dimensional manner, and the processes of data collection also tend to be diversified, so the investigating method of enterprise digitalization also needs to be based on a multidimensional deconstruction perspective with reasonable logic. In addition, the relationship analysis between research objects should be guided by multi-source heterogeneous data, and should inform the observers what the real functional relationship is based on the inherent information on the data. Therefore, research on how enterprise digitalization affects green innovation performance should be driven by multidimensional data. Specifically, this study may consist of the following three parts: First, in order to investigate and deconstruct enterprise digitalization with a reasonable logic, we use enterprise production and operation as the perspective to deconstruct enterprise digitalization into various digitalization features according to different types. On this basis, we use public data including annual reports to achieve objective and accurate measurement of the above-mentioned digitalization features. Second, the large volume of data makes it necessary to employ techniques such as clustering to extract information and hidden patterns (10). Within the principle of similarity within a group and dissimilarity between groups, we use the method of clustering analysis to group the data of enterprise digitalization features into several clusters, thus distinguishing different digitalization transformations of enterprises, and then carry out the remaining research on this basis. Finally, on the basis of distinguishing different digitalization transformations, we use the decision tree algorithm to continue to mine the deeper information between the research objects (11), so as to explore which digitalization features' configuration is the most conducive to enterprises looking to carry out green innovation under the circumstances of digitalization transformation belonging to different enterprises, and thus explain the complex impact of enterprise digitalization on green innovation performance.

This study has the following two contributions for the understanding of digitalization and green innovation. (1) From the perspective of production operation, enterprise digitalization is deconstructed into various features, and uses multi-source heterogeneous objective data to quantify these features. This deconstruction idea can not only satisfy the research paradigm which is conducted by multi-features, but also aggregate the above features into a digitalization indicator, thus providing a more comprehensive measurement method for digitalization-related research and helping enterprises to understand their own digitalization maturity. (2) We apply a relatively new research paradigm of data-driven analysis (DDA), which may be a complement to current prevailing paradigms. This paradigm uses popular machine learning algorithms including the cluster analysis and the decision tree model to conduct research on the basis of multi-source heterogeneous data, so that it can more fully explore the complex impact of enterprise digitalization on green innovation performance. (3) Based on the conclusions of our research, enterprises can judge their own digitalization capabilities and find suitable digitalization transition ideas for improving green innovation performance, so as to effectively help enterprises to be more innovative and sustainable in the context of a digital economy.

The remainder of this paper is organized as follows. In Section 2, Theories, cases, and quantitative studies related to digitalization and green innovation will be presented. In Section 3, research process, deconstruction ideas of enterprise digitalization, and data source and processing are proposed. In Section 4, the analytical methods used in this research are introduced in detail. In Section 5, the results of the data analysis are clearly displayed. In Section 6, we put forward the conclusions and enlightenments, analyze the shortcomings of this research, and figure out the directions of future research.

## 2. Related literature

As green, intelligent, and other characteristics have become the development needs and trends of future industry (12), enterprise digitalization and green innovation have gradually become the current research hotspots. In this study, the performance of enterprise green innovation is both the foothold and the dependent variable, because we want to understand how sustainable and high-quality development can be promoted. What kind of digitalization transformation the enterprise undertakes is the antecedent variable of the research, so the literature review is mainly carried out with this logic.

The literature review includes four parts: (1) Theoretical analysis—to explore why enterprises choose digitalization transformation and the impact of digitalization on green innovation. (2) Case analysis—analyze the impact of digitalization on green innovation in a real-world situation. (3) Quantitative research - review the current work on quantitative analysis and lay the foundation for this research. (4) Literature review - discuss the above contents and analyze their possible defects.

### 2.1. Theoretical analysis

Whether and how an enterprise decides to undergo digitalization transformation depends on its mindset (13). In the era of information explosion, the mindset of enterprises depends on the evaluation of past, present, and future situations, and also on the thinking of various factors (14), especially for the multiple benefits that digitalization may bring. For example, a decision to embark on digitalization transformation may be based on an enterprise's flexibility to respond to external circumstances. It is generally believed that a higher digitalization maturity of an enterprise means more flexibility and fluidity. Therefore, high digitalization maturity can enable production and operations to continue in the face of the crisis brought by the external environment, which is an advantage that cannot be obtained in counterparts with lower maturity (15). Digitalization will continue to ensure uninterrupted business operations, especially when today's enterprises are still facing great uncertainty in the external environment such as COVID-19 (16). In addition, an enterprise's decision to take digitalization-related action may also be driven by the ability of digitalization to ease financial barriers. Research has found that the adoption of cloud-based solutions may help enterprises ease financial barriers, which are more pronounced in large enterprises (17). And the development of scale is the pursuit of most enterprises, which will also improve the feasibility of enterprises to make digitalization-related decisions. Moreover, a decision about whether to undergo a digitization transformation may be related to its impact on innovation activities. According to the theory of entrepreneurial education, entrepreneurship is about taking action on opportunities and ideas and turning them into value for others. Nowadays, innovation activities have been dominated by digitalization (9), and digitalization technologies have become important factors in increasing entrepreneurship (18). Enterprises can seize or develop new knowledge and opportunities with the help of digitalization technologies, and turn these into effective value. Ample evidence shows that digitalization has a positive impact on the innovation ability of enterprise (19). For instance, the establishment of a digitalization platform will enable enterprises to explore more suitable innovation opportunities, making it easier to generate new ideas, knowledge, and solutions (20). As a kind of enterprise innovation activity, green innovation will benefit from the digitalization transformation of enterprises. All of the above evidence points to the benefits of digitalization transformation, so there are sufficient reasons for enterprises to take digitalization action.

The impact of digitalization on the innovation component of green innovation has been discussed above. And digitalization also has a positive effect on the green part of the green innovation. The advancement of digitalization technology has enabled the dissemination of green concepts such as sustainable development at a faster rate. For example, narratives such as “climate change is a hoax” can spread very quickly due to the networked nature of our social media existence (21). Given this change, there has been a positive change in enterprises' attitudes toward these green concepts at the cultural level. This has led to multi-stakeholders, including enterprise managers, seeking to bring about meaningful change in current business practices, with a focus on social and environmental well-being (22). In addition, the application of digitalization technology will make it easier for external stakeholders to verify whether enterprises are practicing green concepts. For instance, the platform built by an enterprise based on blockchain technology will make it more transparent, so as to show the enterprise's circular economy concept and sustainable strategy to the partners in the supply chain (23, 24). This kind of commitment to practice green concepts usually means that it is easier for enterprises to gain external trust. As one of the green concepts, green innovation will also accompany the gradual upgrading of these digitalization technologies to spread its benefits to the society for sustainable development, which will naturally make it valued in the process of organizational practice.

### 2.2. Case analysis

In a realistic scenario, there are many specific cases that can reflect the impact of digitalization on green innovation performance. In China, traditional enterprises represented by industrial enterprises are the focus of green innovation. These enterprises can embed digitalization technologies and platforms such as industrial robots and 3D printing into the entire production and service process of traditional manufacturing enterprises according to their own needs, thereby promoting digitalization transformation to improve green innovation performance (25). Taking the ferrous metal processing industry in Zhejiang Province as an example, the deepening of the digitalization transformation will help such enterprises to free themselves from the influence of excessive production capacity and prevent them from becoming recession-type enterprises again. A typical representative of such enterprises is “Hang Zhou Iron and Steel,” whose innovation frequency has also increased significantly in the process of digitalization transformation. Without affecting production efficiency, it can effectively get rid of excess capacity and ensure sufficient innovation efficiency. This phenomenon reflects that the green innovation efficiency of such enterprises has been effectively improved with the deepening of the digitalization transformation process.

Additionally, He et al. (26) found the following information through the investigation of 11 Chinese industrial enterprises covering the pharmaceutical industry and the automobile industry: digitalization technology empowers management perception, enterprise competitiveness, data and information elements, and enterprise resource utilization efficiency, green product design, process digitalization, and other intermediary roles affect the evolution of enterprise green strategy. This will also ultimately promote green innovation for enterprises.

### 2.3. Quantitative research

Some scholars have also discussed the impact of enterprise digitalization on green innovation from the research process of quantitative analysis, and have drawn relevant conclusions. El-Kassar and Singh (27) conducted research based on the survey data of 215 enterprises and found that the implementation of big data technology can affect the green innovation activities of enterprises and enable them to further gain a competitive advantage. In addition, Yang et al. (28) used Chinese manufacturing enterprises as a research sample and found that the intelligence has a significant promotion impact on green innovation performance. However, their studies both lack a deep analysis on the mechanism of enterprise digitalization affecting green innovation, and the impact path of digitalization on green innovation has not been clarified in detail. Dou and Gao (29) used matching data of Chinese provincial and manufacturing enterprises as samples to conduct research, and believed that regional digitalization has an inverse U-shaped nonlinear impact on green innovation of enterprises. But it is difficult for their research to use the micro or macro perspective of digitalization to explore how it affects green innovation performance, and it is difficult to propose specific ideas about companies on how to practice digitalization transformation to improve green innovation performance. Li and Shen (30) used annual reports to measure the level of digitalization enterprises, and found that enterprises with advanced digitalization transformation have more potential to carry out green innovation. However, it may not be comprehensive enough to investigate the way of enterprise digitalization only from the annual report, so it is worth adding more perspectives to improve. In addition, Wei and Sun (31) also used a questionnaire method to investigate the digitalization of enterprises as comprehensively as possible, and concluded that digitalization can help promote green innovation, but this method requires attention to the reliability of sample sources and the reproducibility of experimental data.

### 2.4. Literature review

Through retrospect of theoretical derivation, case analysis, and quantitative research, there are sufficient reasons to believe that it is inevitable for enterprises to take digitalization-related actions, and the relationship between digitalization and green innovation is also possible. Therefore, it is meaningful to carry out research about the impact of enterprise digitalization on green innovation performance. However, there are still some aspects for improvement, which will be realized in this study: (1) Deriving the impact of digitalization on green innovation only from theoretical deductions seems insufficient, which needs to be supplemented by new evidence from the real-world; (2) Although using the method of case analysis can reflect the specific situation of one or more enterprises and has reference significance, it cannot pay attention to the various heterogeneity differences between enterprises, especially between digitalization; (3) How to reasonably construct and accurately measure enterprise digitalization is the basis of carrying out quantitative analyses, but the current research has the problems of diversification of data sources and inability to unify inspection methods, which may hinder the scholars in carrying out digitalization-related research and enterprises to understand their digitalization competitiveness, so it is necessary to propose a more rigorous and comprehensive enterprise digitalization deconstruction logic; and (4) Most of the existing quantitative research was carried out from an integrated perspective, which only explored the simple impact relationship between enterprise digitalization and green innovation. Therefore, it is difficult to analyze the complex impact of digitalization on green innovation and to provide practical advice for enterprises.

## 3. Research process and research data

In order to successfully complete the research objective, this section contains the following content: the general research process, the deconstructing idea and result of enterprise digitalization, the quantification of green innovation performance, and the source and processing aspects of data. These are the foundational work to explore the complex impact of enterprise digitalization on green innovation performance, so an introduction to them is necessary.

### 3.1. Research process

**Figure 1:**
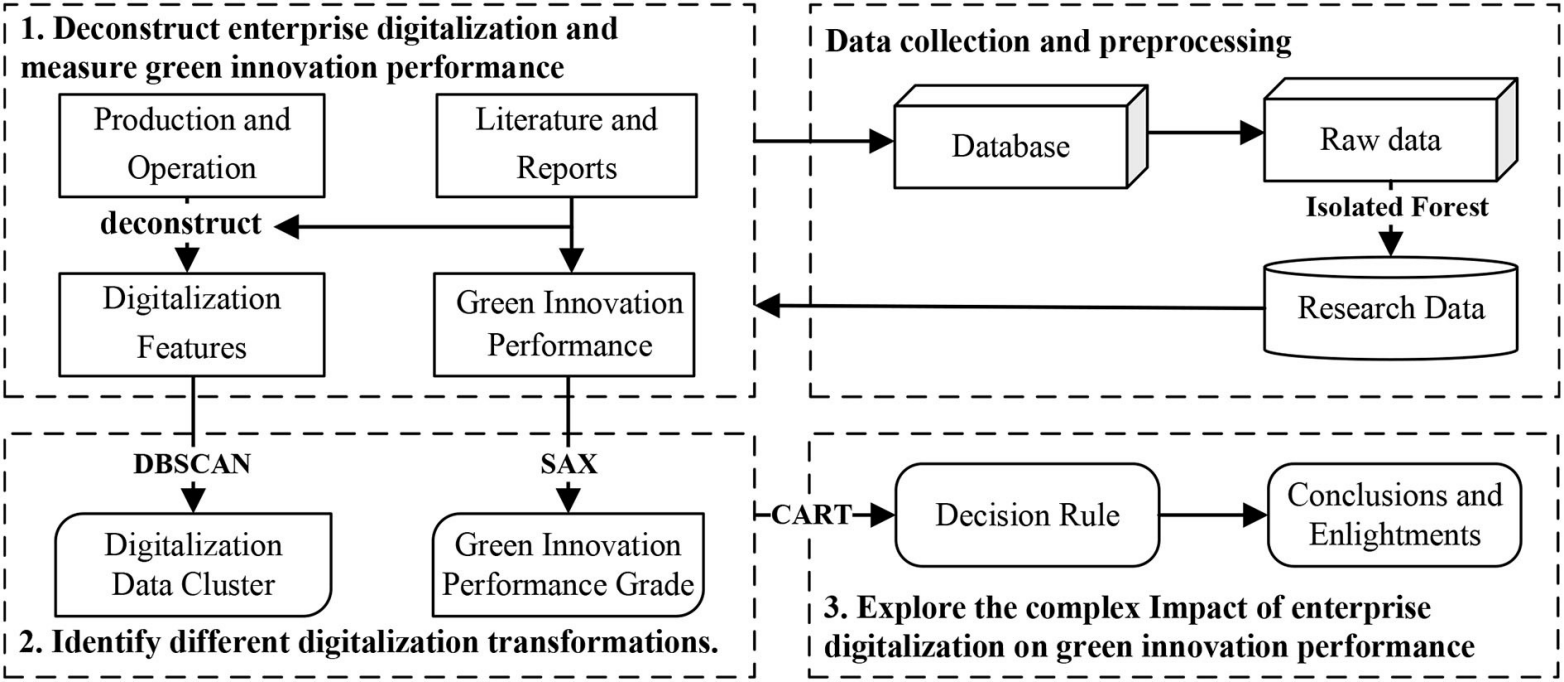
Research process.

Objects in the world usually need to use multiple dimensions or features depicted together (32), and the exploration of relationships between various objects should also be based on multi-source heterogeneous data, so the impact of enterprise digitalization on green innovation should also be carried out based on this idea. In addition, the complex means that enterprise digitalization does not only have a positive or negative impact on green innovation performance by a single variable, but also has a comprehensive impact on green innovation performance of different feature configurations. As shown in Figure 1, this research will be conducted in three parts to analyze the complex impact of enterprise digitalization on green innovation under multiple features, and to solve the problem of how enterprises should practice digitalization transformation to effectively improve green innovation performance.

(1) Deconstructing enterprise digitalization and measuring green innovation performance. Enterprise digitalization should be depicted by using multiple features, which requires a reasonable deconstruction method of enterprise digitalization. By consulting relevant literature, we find that it is logical to take the production and operation of enterprises as a deconstruction perspective. Enterprise digitalization can be decomposed into multiple features in several dimensions on the basis of production and operations. Similarly, the method of green innovation performance also can be identified by reviewing literature. Finally, we obtain the original data of digitalization features and green innovation performance from databases, and remove the outliers by using the Isolation Forest algorithm to obtain the data resources required for the research.
(2) Identify different digitalization transformations. In order to carry out specific analysis on specific problems, the digitalization features data are aggregated into certain clusters with the principle of similarity within the group and dissimilarity between the groups. On this basis, each cluster is appropriately named according to the features' distribution, thereby identifying different types of digital transformation. In addition, green innovation performance is also discretized into different grades, and combined with the clustering results of enterprise digitalization features to analyze the impact of different digitalization transformations on green innovation performance.
(3) Explore the complex impact of enterprise digitalization on green innovation performance. In order to continue to analyze the complex impact of enterprise digitalization on green innovation performance on the basis of identifying the digitalization data distribution of different enterprises, we take the obtained various digitalization features as conditional attributes, take the grade of green innovation performance as the decision attribute, and use the decision tree algorithm to mine the decision rule. Therefore, we can analyze what digitalization transformation path is conducive to the improvement of green innovation performance, and put forward corresponding conclusions and enlightenments accordingly.

### 3.2. Digitalization features and green innovation performance

At present, the methods of investigating the enterprise digitalization are diversified and have not yet reached a unified state, but there are only two ways: objective and subjective. For example, using text mining methods to analyze the information related to enterprise digitalization from annual reports (30), by only using the annual report, can reflect limited information of the enterprise, and it has a certain degree of subjectivity because it is written by the enterprise itself. In addition, some scholars use questionnaires to collect enterprise digitalization data for research, but the reliability of the questionnaire data sources and the possibility of repeating the experiment need to be discussed (31).

Production operation can be a reasonable research perspective for deconstructing enterprise digitalization. When discussing digital transformation, Berman (33) and Van Veldhovenand and Vanthienen (34) believed that there are many ways for enterprises to implement digitalization transformation, including enterprise operations. As an important part of the enterprise operation, production operation focuses on the value-added to the enterprise value chain, including the three basic dimensions of input, output, and management, which is a more coherent and comprehensive inspection logic for enterprises. Additionally, as digitalization transformation progresses, enterprises may change the approach they create and appropriate value (35), and generate effective insights or application patterns that cannot be emerged by traditional production and operations (36), which can be reflected in following examples. First, digitalization technology represented by cloud computing allows enterprises to virtualize resources in a dynamic way, and also provides the possibility for enterprises to obtain resources when needed, avoiding the sunk costs and only paying for the resources actually used (37), and reduce the real cost and psychological cost of production operation from the input side. Second, digitalization technologies such as the Internet of Things (IoT) help enterprises map the situation of work objects to the Internet in a digitalization way, so as to remotely track work orders, determine execution status, and collect feedback data and information in real time (38), so that the production operation of the enterprise can be managed and controlled in a relatively precise way. Third, the application of information and communications technology (ICT) and other similar technologies can realize process automation in production, which not only eliminates the redundancy and waste in the production process (39), but also is one of the main ways to change the energy consumption pattern of the manufacturing process (40), so as to achieve the maximum output with the minimum input, and improve the efficiency on the output side of the production operation. In short, the three dimensions of production operations are logical enough to cover as many situations as possible, and also to compensate for the above-mentioned shortcomings. Nowadays, enterprise digitalization has penetrated into production operation, so how much digitalization information an enterprise contains in the three elements of production and operation can be regarded as a reasonable way to investigate the digitalization of the enterprise.

Based on above research, it is believed that production operations are taken as the investigation perspective, and deconstruct enterprise digitalization from the three dimensions of input, output, and management. However, it is still difficult to accurately cover all information only from the above three dimensions. For example, there are various types of input including innovation input or asset input. Therefore, it is necessary to continue to decompose the above dimensions according to different types, and finally obtain the six enterprise digitalization features shown in Table 1.

**Table 1:** The implications of digitalization features.

**Dimensions**	**Features**	**Quantitative basics**	**Method**
DI	DAI	Intangible assets and fixed assets	Text classification and EWM
	DOI	Operating cost	
	DII	Development expenditure	Text classification
DO	DOO	Operating income	
	DIO	Patent	Automated crawling
DM	DM	Annual report	TF-IDF

As shown in Table 1, there are dimensions, features, quantitative basics, and method in the header. The dimensions include digitalization input (DI), digitalization output (DO), and digitalization management (DM), which can also be regarded as primary indicators. The features are a continuous decomposition of the above three dimensions, including digitalization asset input (DAI), digitalization operating input (DOI), digitalization innovation input (DII), digitalization operation output (DOO), digitalization innovation output (DIO), and digitalization management (DM), which can also be regarded as a secondary indicator. The quantitative basics are the data source for quantifying the above features, including various objective data such as annual reports and so on. The methods are how to convert these objective data into research data that can be analyzed, which are all popular in the academic world.

**Digitalization input (DI)**: The actual expenditure and investment of the enterprise on digitalization. There are three features in this dimension, namely digitalization asset input (DAI), digitalization operating input (DOI), and digitalization innovation input (DII).

**Digitalization asset input (DAI)**: The more an enterprise invests in digitalization assets, the more it can reflect the actual application of digitalization. Existing research found that the response of incumbents to digitalization elements such as technology may differ depending on the resources or assets that need to be mobilized (41). Therefore, an enterprise's investment in digitalization-related assets should be included in the investigation process of enterprise digitalization. As enterprises have more digitalization assets, they will have stronger knowledge integration and communication capabilities, which will have a mechanism effect on green innovation performance (42). The measurement process of this feature is relatively complicated, and it is mainly divided into the following steps: First, based on digitalization-related keywords including digitalization, Internet, and Intelligence, manually pick items related to digitalization from intangible assets and fixed assets belonging to financial statements. Second, the screened intangible assets and fixed assets are vertically aggregated according to the enterprise and year, and divided by the total assets of the enterprise to achieve de-unitization, so as to improve the comparability of enterprise data. Third, according to the data structure of the processed intangible assets and fixed assets, they are weighted by the entropy weight method (EWM) and summed horizontally on this basis, where the weight of intangible assets is 0.28 and the weight of fixed assets is 0.72. After the above steps, the measurement process of the feature is completed.

**Digitalization operating input (DOI)**: In the process of analyzing the production operation of enterprises, the operating cost should be given attention. Therefore, when deconstructing the digitalization of enterprises from the perspective of production operation, it is necessary to analyze the operating cost as one of the features of the input side. Digitalization operating input is the consumption and investment of enterprises in daily operating activities because of digitalization-related things. It is similar to digitalization asset input, which can reflect the application of digitalization, and may also affect green innovation through similar mechanisms. The quantification of this feature also needs to be based on digitalization-related keywords, manually pick the digitalization-related items from the operating costs of the financial statements. The above items are then aggregated vertically by enterprise and year, and divided by total assets to enhance comparability between data.

**Digitalization innovation input (DII):** Scholars have identified that the importance of innovation for the enterprises digitalization transformation (43, 44). Both digitalization innovation and green innovation belong to enterprise innovation activities, and there are certain similarities between them. In view of this similar connection, the emphasis on digitalization innovation by enterprises may have an additional impact on other activities that belong to the same field of innovation, including the green innovation performance, resulting in the “ripple effect” (45). Therefore, when conducting research about digitalization, it is necessary to add the consideration of digitalization innovation, so as to avoid ignoring important variables. Digitalization innovation input is the actual expenditure of enterprises on digitalization innovation, and its measurement process is also similar to the other two features of digitalization input. This feature requires manual selection of digitalization-related items from R&D expenditures in financial statements, then vertical aggregation by enterprise and year, and division by total assets to ensure comparability.

**Digitalization output (DO)**: The output obtained by the enterprise in the process of production and operation due to digitalization. There are two features belonging to this dimension, namely digitalization operation output (DOO) and digitalization innovation output (DIO).

**Digitalization operation output (DOO)**: In the process of analyzing the production operation of enterprises, operation income should also be given attention. Digitalization operation output is the output obtained by the enterprise in the daily business activities due to the digitalization of related things. Therefore, when enterprises attach importance to this feature, they can avoid warehouse backlogs and promote green innovation performance in supply chains and logistics chains. The measurement of this feature is similar to digitalization asset input, which is also necessary to manually pick digitalization-related items from the operating income of financial statements based on digitalization-related keywords. After completing the above steps, we aggregate vertically by enterprise and year, and then divide by total assets to enhance comparability of data.

**Digitalization innovation output (DIO)**: Also due to the existence of the “ripple effect,” when investigating the enterprise digitalization, it is also necessary to consider the digitalization innovation output, and analyze its impact on the green innovation performance. The measurement of this feature is relatively simple, which is to select the application number of digitalization-related patents in a certain year as the measurement basis for this feature. The acquisition method of this feature data is to design a python automation program and crawl from the patent database based on the digitalization-related keywords.There are several categories of Chinese patents, including patents for innovation, utility models, and industrial designs, and these patents have different values, so it is necessary to give different weight to them when measuring this feature.


(1) $$\begin{array}{l} DIO^{i}=\overset{3}{\underset{j=1}{\sum }}Dw_{j}^{i}Dp_{j}^{i} \end{array}$$

Where *DIO*^*i*^ is the digitalization innovation output belonging to the *i*-th enterprise. $Dp_{j}^{i}\left(j\in \left(1,2,3\right)\right)$ represent patents for innovation, utility models, and industrial designs related to digitalization in order. $Dw_{j}^{i}\left(j\in \left(1,2,3\right)\right)$ are the weight assignment corresponding to the above three type of patents, which are 0.5, 0.3, and 0.2, respectively.

**Digitalization management (DM)**: There is only one feature with same name in this dimension, which reflects the relationship with digitalization when making management decisions. According to the mechanism of “mindsponge,” enterprises absorb new cultural and ideological values in a multi-dimensional environment, which will help them adapt to the new environment and identify better development opportunities (46, 47). The concept of digitalization management proposed here is to reflect the performance of this mechanism in digitalization. Enterprises' emphasis on digitalization management can mobilize emotions from top to bottom, and promote the digitalization maturity from the perspective of personnel psychology or soft culture, thereby accelerating the digitalization transformation process of enterprises to adapt to the environment of a digital economy. The annual report can reveal the enterprise's review of the past and expectation of the future (48), which can show the enterprise's reflection and outlook on the management methods. Therefore, the measurement of this feature can be based on the enterprise's annual report for the sentiment analysis. The specific quantification steps for digitalization management are as follows. First, based on the classification standards of the *Digital economy and its core industries statistical classification (2021)* published by the National Bureau of Statistics in China, establish a digitalization keyword dictionary, including a total of 166 digitalization-related words including big data, cloud computing, and so on. Second, take the annual reports of all enterprises in the same year as the corpus, and use a term frequency inverse document frequency (TF-IDF) algorithm to calculate the digitalization sentiment weights for the above words according to each year. Third, the sentiment weights of the above keywords are added up according to the year and the enterprise, so as to obtain the raw data of digitalization management.

**Green innovation performance (GIP)**: This is the final output of enterprises in green innovation. Academia's focus on green innovation performance mainly focuses on technological innovation output, so this measurement is based on the cumulative realization of the number of green patent authorizations obtained by enterprises within a certain period of time. In addition, the World Intellectual Property Organization (WIPO) stated in *IPC Green Inventory* that the identification of green patents is based on the IPC classification number, while industrial designs do not own an IPC number, so green patents mainly focus on the invention authorization type and the utility model in our research. The measurement formula of green innovation performance is as follows:


(2) $$\begin{array}{l} GIP^{i}=\overset{3}{\underset{j=1}{\sum }}Gw_{j}^{i}Gp_{j}^{i} \end{array}$$

Where *GIP*^*i*^ is the green innovation performance belonging to the *i*-th enterprise. $Gp_{j}^{i}\left(j\in \left(1,2\right)\right)$ represent patents for innovation and utility models related to green innovation in order. $Gw_{j}^{i}\left(j\in \left(1,2\right)\right)$ are the weight assignment corresponding to the above two green innovation patents, which are 0.6 and 0.4, respectively.

### 3.3. Data source and processing

The production and operation of an enterprise needs to adjust its expectations for the future in a timely manner according to the current situation, and formulate appropriate goals to implement changes. The topic of this research is what digitalization transformation steps should enterprises take to help improve green innovation performance, which includes the needs of companies looking forward to the future based on the current situation. The analysis of the current situation of the enterprise is usually based on the year-end situation, so the data used to measure the digitalization features are the ending value for the same period. In addition, because green innovation performance is the foothold of this research, the number of green patents authorized are used as the basis of measurement, with a lag of one period to ensure logical consistency.

According to the quantitative requirements, the data types used in the research include financial statements, annual reports, and patent data. The financial statement data comes from the China Stock Market & Accounting Research Database (CSMAR) and RESSET database, the annual reports come from CNIFO, and the patent data is jointly acquired from the China National Knowledge Infrastructure (CNKI) and the China Research Data Service Platform (CNRDS). These databases are relatively authoritative and widely used by academia in China. Since the research focuses on green innovation performance, it mostly focuses on real economy enterprises, so the scope of enterprises is locked in industrial enterprises. In addition, the time span selected for our research is from 2016 to 2020, which is the year when the concept of digital economy and green environmental protection received more attention. This period is also the peak period when China launched a combination of policies to promote innovation and sustainability.

After the raw data was processed by the above quantification process, it needed to be matched with enterprises and years. In order to ensure the reliability of the research results, the null values were first removed from the original data. Then the isolation forest algorithm also removed abnormal data on the above basis. Combining the above steps, 8,154 objects with matching enterprises and years were finally obtained.

## 4. Research method

As shown in the research ideas of Figure 1, the purpose is to analyze how enterprises should practice digitalization transformation to effectively improve green innovation performance from the perspective of production operation, and to analyze which digitalization features' configuration is most conducive to improving green innovation performance. Therefore, it is necessary to use the following methods to assist in implementation.

### 4.1. Density based spatial clustering of applications with noise

**Figure 2:**
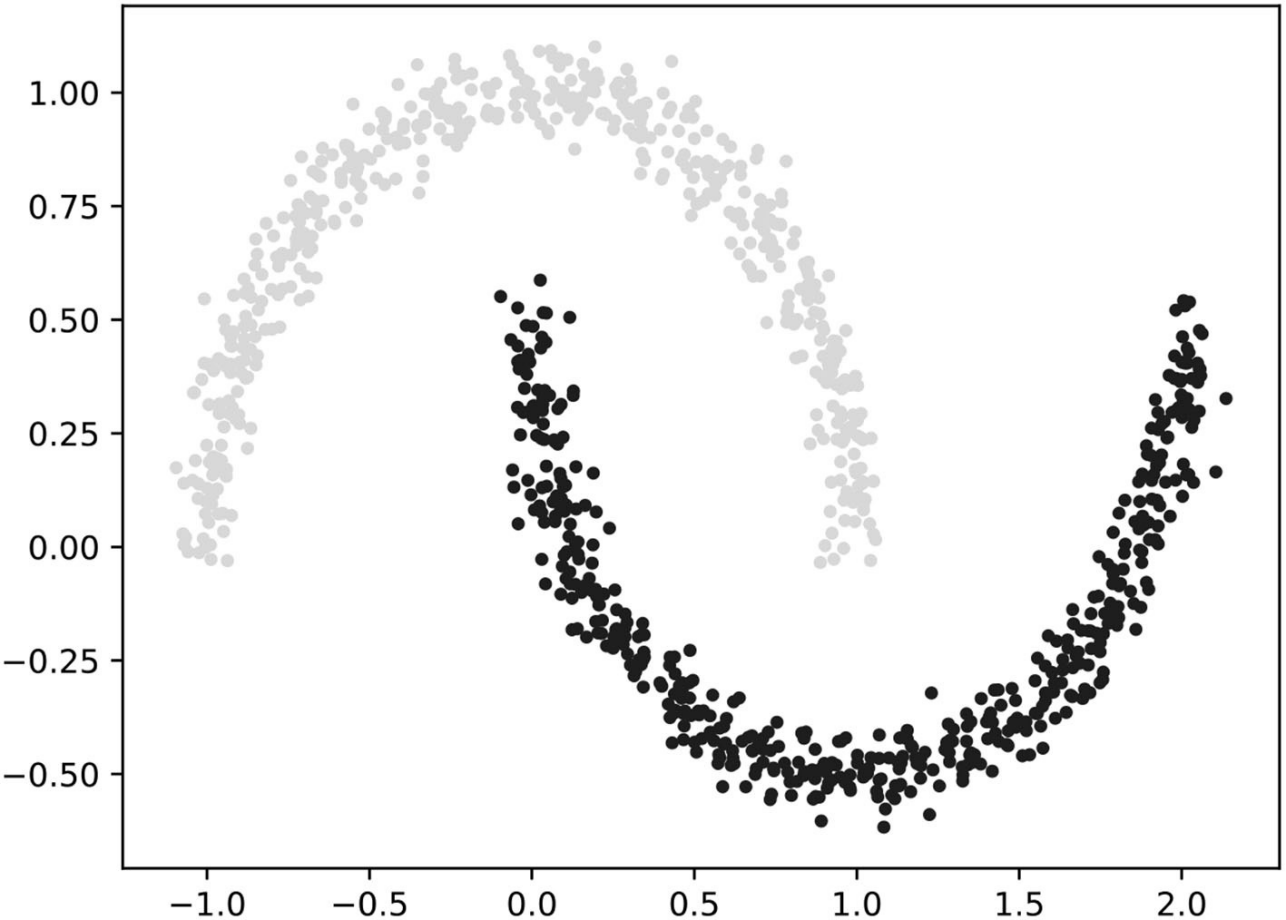
Clustering results of DBSCAN.

In the era of digital economy, a large amount of data needs to use clustering algorithms to assist in mining the hidden information. Nowadays, clustering methods have been widely used in time series analysis (10), bibliometric analysis (49) and other fields. For enterprise digitalization data, clustering algorithms should also be used first to avoid missing some deep knowledge. In other words, various enterprise digitalization transformations may have great differences, which means the results of the analysis corresponding to different digitalization features' configuration may not be consistent, so it is difficult to draw targeted conclusions if all the research data is analyzed at the same time. If there are consistent parts of the analysis results obtained, it may also ensure the robustness of the final conclusions. In order to successfully complete the clustering work, we use the density-based spatial clustering of applications with noise (DBSCAN) clustering algorithm. This algorithm needs to preset two parameters, *eps* (epsilon, which means the radius) and *MinPts* (the minimum number of points in this radius), so as to determine the minimum number of samples existing in a certain range to assume a distribution density threshold, and to cluster data points based on the density range. As shown in Figure 2, the DBSCAN clustering algorithm can not only cluster dense data sets of any shape, but also find outliers during clustering to ensure the results are not biased (55).

The algorithm randomly selects an unprocessed point from research data and judges whether the number of adjacent points within *eps* belonging to it is greater than *MinPts*. If the point satisfies this condition, it is considered as a core point and the points within the density range are clustered into a cluster; otherwise the point is regarded as a border point (non-core point) and skipped. Additionally, this algorithm continues to search for the other point until all data is traversed and obtains all clusters. After the research data is processed by this algorithm, it can be effectively distinguished according to the features data distribution, so as to ensure that the analysis results have sufficient pertinence and practical value.

### 4.2. Symbolic aggregate approximation

As a decision attribute, green innovation performance (GIP) needs to be classified in advance. Since the research data is numerical data, the SAX algorithm is the algorithm of choice for symbolizing and discretizing the data to objectively obtain a green innovation performance rating. This algorithm was originally used for time series dimensionality reduction (50), but it can still be used for classification of feature data due to its ability to accurately distinguish data distributions.

There are three main steps in this algorithm. First, the grading work needs to be carried out under unified standards, so the loaded data must be standardized. Second, the standardized data needs to be dimensionally divided into sub-intervals of equal length by using the method of Piecewise Aggregate Approximation (PAA), and replaced by the mean of each sub-interval (51). Third, this algorithm should preset the division space number α to determine the division coefficient, and divide the data into different state identifiers to symbolize the data.

**Figure 3:**
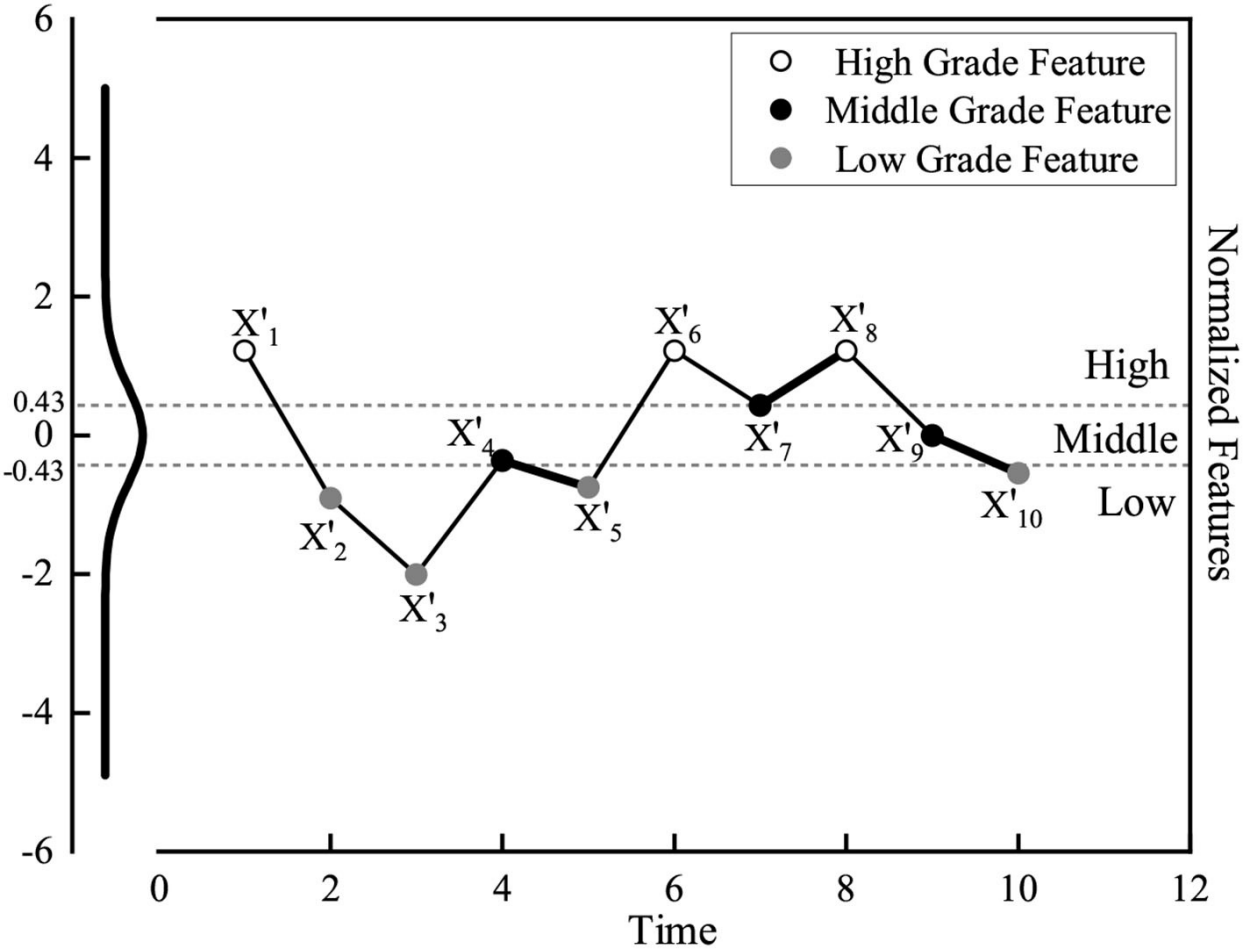
The grading results of SAX.

As shown in Figure 3, it is assumed that there is a normalized time series feature that is about to be graded by the SAX algorithm, and when α is set to 3, the division coefficient is locked to 0.43 and -0.43, and the feature is divided into three grades. Therefore, it is believed that the SAX algorithm can also complete the grading work of green innovation performance.

### 4.3. Classification and regression tree

In order to find the ideas about improving the green innovation performance (GIP) from the digitalization data clusters of different enterprises, and to know what kind of digitalization transformation processes enterprises should implement to help improve the green innovation performance (GIP), the CART decision tree algorithm is used to extract the decision rules and obtain the complex relationship between the former and the latter. As a traditional classification algorithm, the CART decision tree algorithm can effectively identify the optimal feature configuration for a certain state (52). Compared with other decision tree algorithms such as ID3, CART is a classification and regression algorithm based on a binary decision tree, which is suitable for numerical data (11), and is also more convenient to interpret. The CART decision tree algorithm uses the Gini coefficient (*Gini*) to reflect the impurity of the feature data. The smaller the *Gini*, the lower the impurity. It also uses the conditional Gini coefficient Gini(*D*∣*k*) to reflect the feature importance and alternative priority of the conditional attribute to the decision attribute. The smaller the coefficient, the higher the importance and alternative priority of the feature. During the operation of the algorithm, the cut value (*k*) with the smallest Gini(*D*∣*k*) is selected to generate a binary tree. After each binary tree is formed, the algorithm also calculates the Gini(*D*∣*k*) for the divided parts to continue to generate branches until it can no longer be divided.

Assuming that a cluster *D*^*n*^ clustered by DBSCAN has *K* features, the probability that each value *k* may be taken for these features is *p*_*k*_ , the basic calculation formula of *Gini*:


(3) $$\begin{array}{l} \mathrm{Gini}\left(p\right)=-\overset{K}{\underset{k=1}{\sum }}p_{k}\left(1-p_{k}\right)=1-\overset{K}{\underset{k=1}{\sum }}p_{k}^{2} \end{array}$$

If *k*_*n*_ is all the values *K*_*n*_ of a feature in *D*^*n*^, $D_{2}^{n}$ and $D_{2}^{n}$ refer to the two parts of *D*^*n*^ divided by *k*_*n*_, then the conditional Gini coefficient ($\mathrm{Gini}\left(D^{n}|k_{n}\right)$) is:


(4) $$\begin{array}{l} \mathrm{Gini}\left(D^{n}|k_{n}\right)=\frac{\left|D_{1}^{n}\right|}{\left|D^{n}\right|}\mathrm{Gini}\left(D_{1}^{n}\right)+\frac{\left|D_{1}^{n}\right|}{\left|D^{n}\right|}\mathrm{Gini}\left(D_{2}^{n}\right) \end{array}$$

In the decision tree, there are four concepts, *Path*, *Support*, *Confidence*, and *Lift*, which need to be clarified. Based on the research of Agrawal et al. (53), these concepts have the following information in the decision tree: *Path*, *Support*, *Confidence*, and *Lift*, which represent the following information. (1) The *Path* means the promotion path or decision path, which is also a specific decision rule used to describe the performance of green innovation by feature configuration. (2) *Support* is expressed as the probability of itemsets appearing in the total itemsets, that is, the proportion of the sample size of the decision path to the total number of cluster samples. If the total number of samples is *T* and the sample size of a decision path is *P*, then the support of the path is *Support* = *T*÷*P*× 100%. (3) *Confidence* represents the probability that a specific event occurs under a certain condition, that is, the ratio of the number of samples corresponding to the final classification of the path to the total sample size of this path. If the final classification of a decision path is high, where the number of high samples is *H* and the sample size of this path is *P*, *Confidence* in the case of high-grade classification is *Confidence* = *H*÷*P*× 100%. (4) *Lift* represents the ratio of the probability of a specific event occurring under a certain condition to the probability of the event occurring under the global conditions, so *Lift* of a certain grade can also be regarded as the ratio of the *Confidence* of this grade to the *Confidence* of the total sample. If the final classification of the decision path is high, the high-grade's *Confidence* of this path is *C*, and the total sample high-grade *Confidence* is *O*, then the *Lift* is *Lift* = *C*÷*O*× 100%. *Lift* greater than 1 indicates that the decision path has a positive correlation with a certain state, and the higher the *Lift*, the stronger the positive correlation, and vice versa. For the convenience of presentation, the *Lift* displayed in our research is described according to the final grade of the decision path; if the grade obtained by the path is high, the calculation of the *Lift* is carried out according to the same high-grade.

**Figure 4:**
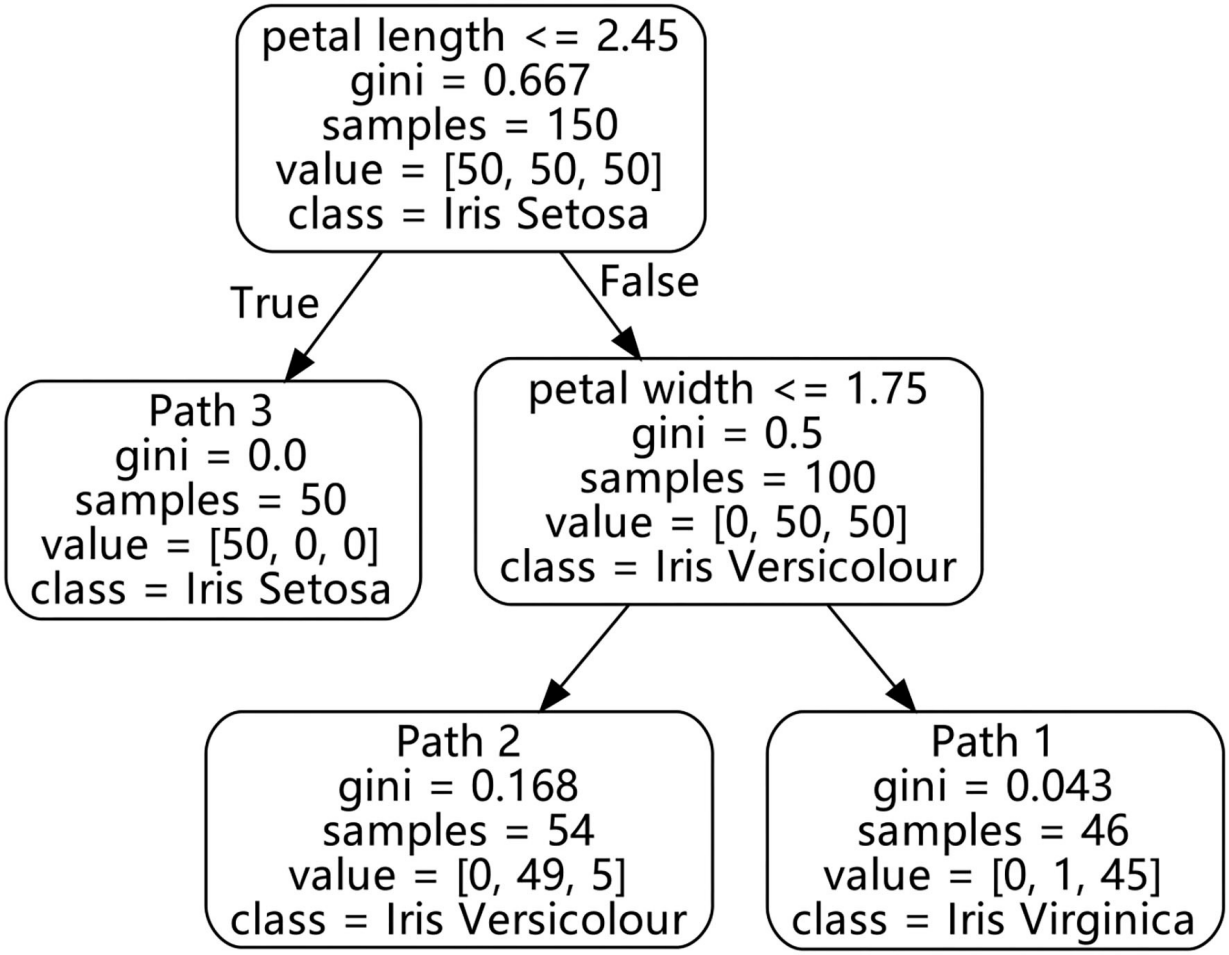
Classification decision tree for iris.

As shown in Figure 4, this algorithm can be used to distinguish different types of irises, which can clearly display the classification results. The path shown in the figure informs the observer that different types of irises can be distinguished by this decision rule. For example, if an iris has a long petal length and a large petal width, it can be considered an Iris Virginica according to *Path*1. The *Confidence* of this path is 97.8% and the *Support* of it is 30.7%, which means that there is sufficient confidence to believe the classification obtained by this path. Further, the *Lift* of this path is 2.94, which means this decision rule has a strong positive correlation with the classification of Iris Virginica.

After the research data is processed by the CART decision tree algorithm, several important features and a classification path combined with these features can be obtained. Based on these rules, the nonlinear combination effects of different digitalization features on the green innovation performance can also be analyzed, so as to provide corresponding analysis results and management implications.

## 5. Green innovation analysis

After identifying the deconstructing ideas of production operations, research data of industrial enterprises, and research methods of machine learning, we combine them for data analysis and continue to explore how enterprises should practice digitalization transformation to effectively improve green innovation performance. Digitalization features data is clustered using the DBSCAN algorithm to identify enterprises with heterogeneous differences in digitalization performance. Using SAX to classify decision attributes, and combining the clustering results, a preliminary analysis was made on the impact of enterprise digitalization on green innovation performance. Furthermore, on the basis of identifying enterprises with heterogeneous digitalization performance and grading green innovation performance, the nonlinear effects of different digital feature configurations on green innovation performance are explored using CART. Combined with the analysis results of clustering and decision tree, we also do a horizontal comparative analysis between different decision trees, and continue to mine the information hidden in the data.

### 5.1. Identify different digitalization transformations

**Figure 5:**
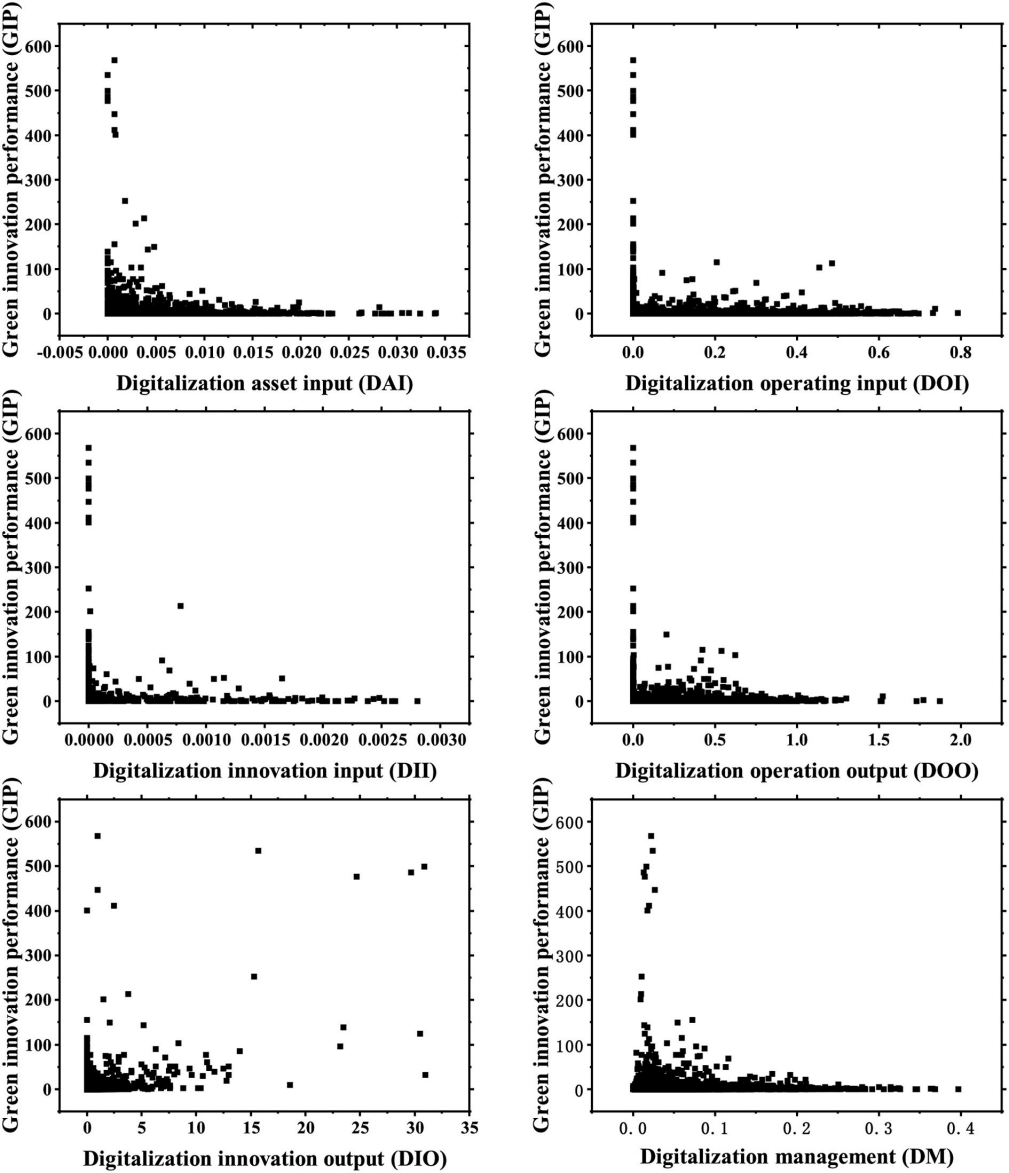
Scatterplot of the relationship between digitalization features and green innovation performance.

For the purpose of verifying that each digitalization feature has an impact relationship on green innovation performance under the perspective of production operation, a scatter diagram of the relationship among all features and green innovation performance is drawn. As shown in Figure 5, there may be correlations among various digitalization features and green innovation performance, indicating that enterprise green innovation performance is not only affected by a single enterprise digitalization feature. Therefore, when discussing the impact of enterprise digitalization on green innovation performance, the analysis should be conducted from the complex perspective of features' configuration.

In order to continue to analyze the complex impact of enterprise digitalization features' configuration on green innovation performance from the perspective of production operation, and to obtain targeted conclusions, it is necessary to cluster enterprise digital data. As mentioned above, the DBSCAN contains two variables, eps and MinPts, that need to be preset. When eps is set to 0.5 and MinPts is set to 100, the research data are aggregated into two clusters with the principle of similarity within the group and dissimilarity between the groups, which is a stable state that is hard to continue subdividing. To verify the accuracy of the clustering results, the elbow method is also used to calculate the silhouette coefficient, and finally arrive at the suitable number of clusters as being two.

**Figure 6:**
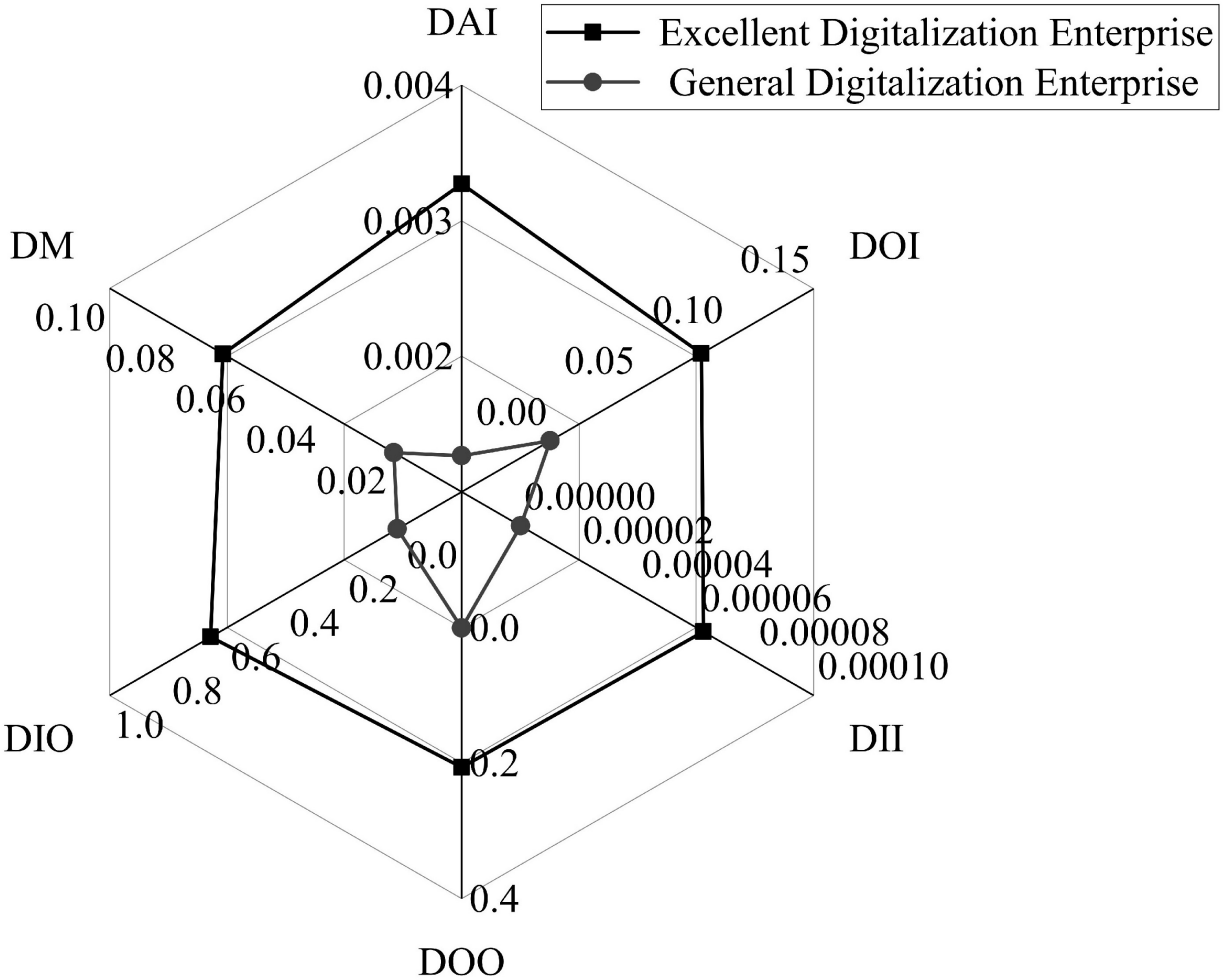
Radar chart of enterprise digitalization features.

Due to the fact that different features groups obtained by DBSCAN may have differences between groups, it is necessary to continue to analyze the clustering results. Table 2 lists the basic statistical information of each cluster, and Figure 6 is a radar chart based on the mean of digitalization features for different clusters. It can be learned from Figure 6 and Table 2 that the clustering algorithm has a significant distinguishing effect on the research data, the obtained results show an obvious bipolar distribution. There are a total of 2626 enterprise digitalization samples in the first cluster, and the shape of the radar chart is close to a regular hexagon, indicating that the distribution of the features of the cluster is relatively balanced. Compared with the other cluster, the data of this cluster is in a more peripheral position, and the descriptive statistical variables of its six digitalization features are also better, so the cluster is named excellent digitalization enterprises. The second cluster has a total of 5,528 enterprise digitalization samples, and its radar chart is closer to a triangle, indicating that the distribution of the features in this cluster is relatively uneven. Compared with first cluster, its radar chart position is closer to the center, and the digitalization features are lower than that of another cluster, so the cluster is named general digitalization enterprises.

**Table 2:** Descriptive statistics table of enterprise digitalization features under clustering results.

**Cluster**	**Construct**	**DAI**	**DOI**	**DII**	**DOO**	**DIO**	**DM**
	Max	0.034	0.794	0.003	1.87	31.000	0.397
Excellent digitalization enterprises	Min	0.000	0.000	0.000	0.000	0.000	0.000
	Mean	0.003	0.086	0.000	0.206	0.656	0.068
	Std	0.004	0.146	0.001	0.269	2.021	0.059
	Max	0.010	0.028	0.000	0.078	0.800	0.094
General digitalization enterprises	Min	0	0	0	0	0	0
	Mean	0.001	0.000	0.000	0.001	0.019	0.019
	Std	0.002	0.002	0.000	0.004	0.090	0.014

In order to analyze how enterprise digitalization affects green innovation performance under different clusters, and to meet the requirements of decision tree algorithm operation data, it is necessary to grade green innovation performance. The specific steps are as follows. First, the data that deviates too much from the normal range is removed to avoid the impact of extreme values on the rank division. The lowest green innovation performances are 0, which belong to the majority, and data with a large deviation from the normal range are all extremely high values, so the final green innovation performance used for grading ranges from 0 to 4.4. Second, the α of SAX is set as 2 to discretize the remaining data, thus making 0.6 the threshold to divide the green innovation performance into two grades: high and low, which means the green innovation performance lower than 0.6 is classified as low, otherwise it is high. Third, all extremely high values which were deleted previously are set to high-grade, thus completing the classification of green innovation performance.

Through the analysis of the clustering results of enterprise digitalization features and the grades of green innovation performance, the following findings can be obtained. There are 1,516 enterprise data with high green innovation performance of excellent digitalization enterprises which accounting for 57.7% of the number in this cluster. In addition, there are 2,199 items of high green innovation performance of general digitalization enterprises which accounting for only 39.8% of the total number in this cluster. Therefore, enterprises with a higher degree of digitalization transformation can usually achieve high green innovation performance, which also means that digitalization has a positive impact on green innovation performance.

### 5.2. The green innovation of the excellent digitalization enterprises

In order to find the promotion paths and feature configurations of different enterprises' digitalization on green innovation performance, and to verify the phenomenon shown by the scatterplot, the CART decision tree algorithm is used to mine decision rules. On the basis of completing the cluster analysis, the 6 digitalization features are taken as conditional attributes and the discretized green innovation performance is used as the decision attribute to respectively construct decision trees for the cluster to extract decision rules. Besides, the decision rule tables and corresponding decision tree diagrams are also drawn to clearly display the decision rules.

**Figure 7:**
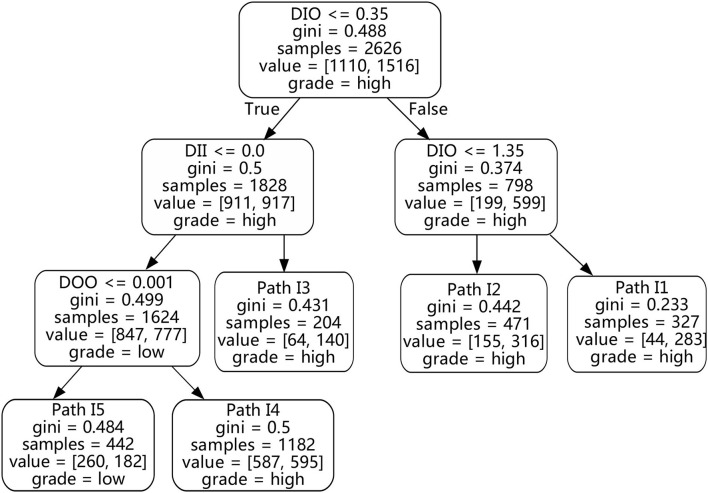
Decision tree for excellent digitalization enterprises.

As shown in Figure 7 and Table 3, the green innovation performance decision rules of excellent digitalization enterprises are mainly positively affected by digitalization innovation output (DIO), digitalization innovation input (DII), and digitalization operation output (DOO) under the perspective of production operation.

**Table 3:** Green innovation performance (GIP) decision rule table for excellent digitalization enterprises.

**Path**	**DAI**	**DOI**	**DII**	**DOO**	**DIO**	**DM**	***Support*(%)**	***Confidence*(%)**	***Lift***	**Grade**
I1	-	-	-	-	> 1.35	-	12.5	86.5	1.50	High
I2	-	-	-	-	(0.35, 1.35)	-	17.9	67.1	1.16	High
I3	-	-	> 0.0	-	≤ 0.35	-	7.8	68.6	1.18	High
I4	-	-	> 0.0	> 0.001	≤ 0.35	-	45.0	50.3	0.87	High
I5	-	-	> 0.0	≤ 0.001	≤ 0.35	-	16.8	58.8	1.29	Low

In excellent digitalization enterprises, digitalization innovation output is the primary and positive digitalization feature of green innovation performance. According to Figure 7 and Table 3, it can be seen that the digitalization innovation output is the highest priority node of the decision tree, which is located at the vertex of the tree. There are two promotion paths in the decision tree with high classifications named I1 and I2, with corresponding *Confidence* of 86.5% and 67.1% in order. This indicates that among the group of excellent digitalization enterprises, enterprises with higher digitalization innovation output are more likely to achieve higher green innovation performance. In addition, the high-grade *Lift* of I1 and I2 are respectively 1.50 and 1.16, which indicate that as the proportion of digitalization innovation output increases, green innovation performance may be promoted accordingly. The *Support* of these two promotion paths are respectively 12.5 and 17.9%, which further support the above analysis results to a certain extent. For excellent digitalization enterprises, their own digitalization transformation process has been relatively advanced, so the activity of paying attention to digitalization innovation output can be regarded as the icing on the cake. Both digitalization innovation output and green innovation performance belong to the output of enterprise innovation activities, so they have certain homogeneity in required resources, knowledge, and other aspects, and also have similarities in the affected steps and mechanisms. Therefore, it can be considered that enterprises that attach importance to the digitalization innovation output are likely to receive a positive response from green innovation performance, resulting in a “ripple effect.”

Digitalization innovation input and digitalization operation output are both positive factors that influence green innovation performance of such enterprises, and are relatively more important than other factors. In I3, when the digitalization innovation input or digitalization operation output is high, there is a possibility for the enterprise to achieve a high-grade in green innovation performance. Although the *Support* of I3 is only 7.8%, the high-grade *Lift* of this promotion path is also 1.18, and the *Confidence* is 68.6%, which is higher than the *Confidence* of the directly connected parent node. The above phenomenon shows that with the promotion of these two digitalization features, the negative impact on green innovation performance due to the low digitalization innovation output can be alleviated. Besides, if digitalization innovation input and digitalization operation output are both low, it will only further aggravate the dilemma of low green innovation performance due to low digitalization innovation output. First, even if the green innovation performance of promotion path I4 is classified as high, and *Confidence* is 50.3%, but only the digitalization operation output in this promotion path is high, and its high-grade *Lift* is 0.87, which is less than 1, this would indicate that there is a negatively correlated influence relationship between this path and the green innovation performance. Second, all features of I5 are relatively low, and the final grade of green innovation performance is also low, with a *Support* of 16.8%, and the corresponding low grade *Confidence* of 58.8%, which is significantly different from the original high-low grade ratio of excellent digitalization enterprises. Moreover, the low grade *Lift* of I5 is 1.29, which is worse than the green innovation performance of I4. Digitalization innovation input is also a type of innovation activity, so enterprises that give resources and attention to digitalization innovation will also exert a positive impact on green innovation. Further, if an enterprise obtains sufficient revenue and financial performance from digitalization, it may be enough to relieve certain financial pressure, thereby helping enterprises to develop and innovate economically, and positively promote green innovation performance. Higher digitalization operation output means that the enterprise is more dependent on digitalization products to obtain income, and this type of product is mostly intangible or virtual, which helps the enterprise reduce the pressure on inventory and the supply chain, thus helping it to improve green innovation performance on the supply chain. In short, these two digitalization features will help the green innovation performance of such enterprises to further improve, thus jointly forming the direction of green digitalization transformation.

### 5.3. The green innovation of the general digitalization enterprises

**Figure 8:**
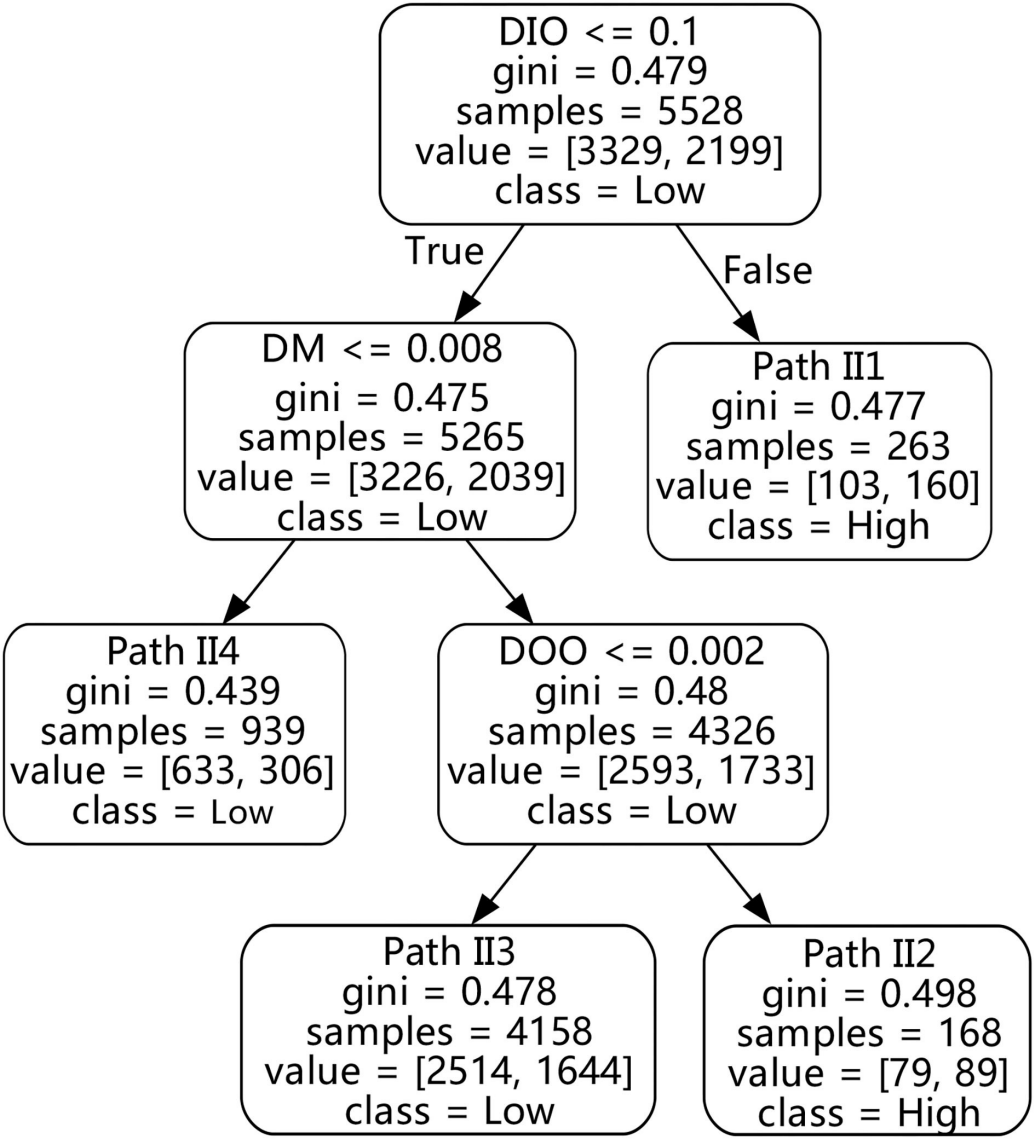
Decision tree for general digitalization enterprises.

To continue exploring the complex impact of digitalization on green innovation performance in the general digitalization enterprise, the same steps are used to build a decision tree. As shown in Figure 8 and Table 4, the green innovation performance (GIP) decision rules of general digitalization enterprises are mainly positively affected by digitalization innovation output (DIO), digitalization management (DM), and digitalization operation output (DOO) under the perspective of production operation.

**Table 4:** Green innovation performance (GIP) decision rule table for general digitalization enterprises.

**Path**	**DAI**	**DOI**	**DII**	**DOO**	**DIO**	**DM**	***Support*(%)**	***Confidence*(%)**	***Lift***	**Grade**
II1	-	-	-	-	> 0.1	-	4.8	60.8	1.53	High
II2	-	-	-	> 0.002	≤ 0.1	> 0.008	3.0	53.0	1.33	High
II3	-	-	-	≤ 0.002	≤ 0.1	>> 0.008	75.2	60.5	1.01	High
II4	-	-	-	-	≤ 0.1	≤ 0.008	17.0	67.4	1.20	Low

In the general digitalization enterprises, digitalization innovation output is also the primary and positive digitalization feature of green innovation performance. Digitalization innovation output is located at the top node of the decision tree, indicating that this feature has the most abundant information and the greatest influence on green innovation performance in this group. There is a decision path II1 in the tree, that when the enterprise obtains higher digitalization innovation output, it will make the final classification of this promotion path high. The high-grade *Lift* of this path is 1.53, indicating that digitalization innovation output has a significant positive effect on green innovation performance. The high-grade *Confidence* of this path is 60.8%, which also significantly increases the proportion of high-grade states of green innovation performance in this cluster. However, the *Support* of II1 is only 4.8%, indicating that these types of enterprises are less likely to achieve high green innovation performance by virtue of this path. For general digitalization enterprises, as long as the feature of digitalization innovation output is high, it is still possible to promote the green innovation performance by virtue of the similarity of input resources and innovation process among different innovation activities.

In this type of enterprise, digitalization management (DM) and digitalization operation output (DOO) are both positive factors that promote the green innovation performance (GIP), and they are also relatively important digitalization features compared with other factors. In other words, if this type of enterprise pays attention to digitalization management and digitalization operation output, it can alleviate the green innovation performance damaged by the lower digitalization innovation output. As shown in Figure 8 and Table 4, there is a promotion path II2 on the left side of the tree where green innovation performance is finally classified as high-grade, in which digitalization innovation output is low, while digitalization management and digitalization operation output are at a high state. Correspondingly, the high state *Confidence* of this path is 53.0%, and the high state *Lift* is also 1.33, which indicate that digitalization management and digitalization operation output have a positive impact on green innovation performance. However, the *Support* of II2 is only 3.0%, indicating that only a few enterprises can rely on this path to achieve high green innovation performance. Combined with the analysis of decision rules of excellent digitalization enterprises, enterprises should boost the overall digitalization process in order to more effectively promote green innovation performance. In addition, there are also two promotion paths for such enterprises, II3 and II4, and the corresponding green innovation performances are all low grades. Their corresponding *Confidence* scores are 60.5% and 67.4%, and the low grade *Lift* scores are respectively 1.01 and 1.20. This phenomenon shows that general digitalization enterprises need to rely on the promotion of digitalization management and digitalization operation output to achieve high green innovation performance. The *Lift* of path II3 is close to 1, which indicates that this path is difficult to change the fact that these types of enterprises were originally in a state of low green innovation performance, but at least it can ensure that the classification of green innovation performance will not be deteriorated. Based on the above analytic result, it can be seen that because of strong digitalization management, the attitude of enterprises toward digitalization transformation will be more positive, and the overall management mindset of enterprises will move toward more digitalization, and become flatter and more transparent. Moreover, a relatively higher digitalization operation output may help reduce inventory to achieve green innovation on the supply chain, and to have more power to promote green innovation performance.

### 5.4. Comparison results of the different digitalization transformations

It is not comprehensive enough to analyze only a single decision tree, so it is necessary to make a horizontal comparison of different digitalization transformations. Through the horizontal comparative mining of the above two sets of decision trees, the following hidden information was also found.

If an enterprise has a relatively excellent digitalization transformation already, it is easier to improve the performance of green innovation, otherwise, the enterprise pays a higher price. In the decision tree of the group of general digitalization enterprises, there are relatively few decision paths with high-grade green innovation performance and the corresponding *Confidence* and *Support* are lower, while in excellent digitalization enterprises the results are the opposite. This phenomenon further shows that enterprise digitalization has a positive impact on green innovation performance, and the positive impact of various digitalization features of enterprises with a higher overall digitalization level will be stronger. With the improvement of the digitalization transformation, the ability of enterprises to perceive the external environment is enhanced, and the ability to seize innovation opportunities is also strengthened (20). The concept of green, as a popular component of the external environment, will be more easily perceived in enterprises with a higher level of digitalization. This type of enterprise is more likely to discover that this is a profitable thing, which will push the enterprise thought process and final decision-making more toward green-related information, and seize the opportunity to improve the green innovation performance.

For enterprises in different digitalization transformation situations, the digitalization features' configurations that help improve green innovation performance are not exactly the same. Digitalization innovation output is the primary digitalization feature that enterprises that want to improve green innovation performance should give positive attention to, and digitalization operation output is another feature that requires positive attention from enterprises. In addition, excellent digitalization enterprises, should pay more attention to digitalization innovation input, otherwise they need to pay attention to digitalization management. In the cluster analysis and previous research, it is found that enterprise digitalization has a positive effect on green innovation performance, which echoes previous research (31). Combined with the analysis results displayed by the decision tree, it is believed that with the further deepening of the digitalization transformation process of enterprises, it may further promote enterprises to improve their green innovation performance. For enterprises with relatively common digitalization transformation, they should first turn their management mindset to digitalization, realize top-down digitalization reform from the management level, and cultivate the digitalization culture of the organization, so as to facilitate the development of green innovation.

Digitalization-related outputs play a more important role in the classification of high green innovation performance. Observing the two decision trees, it is not difficult to find that the two digitalization features related to digitalization innovation output and digitalization operation output are in relatively important positions in the decision tree. However, digitalization input only appears in one feature of excellent digitalization enterprises, which is digitalization innovation input. Therefore, it is speculated that the cost input is immediate, and the effect brought about is not easy to manifest in a relatively short period of time. In other words, digitalization innovation input is important for improving green innovation performance, but it may take a longer time span to show. On the contrary, the time point of digitalization innovation output may be closer to the green innovation performance, so it is more likely to affect the green innovation performance due to the “ripple effect.”

## 6. Conclusions and discussions

In this section, we will summarize the work done and thus present the corresponding conclusions. According to these conclusions, the enlightenment is given to enterprises and governments. Finally, we summarize the gaps in the article and illustrate actions that can be taken by future research.

### 6.1. Conclusions

With the emergence and development of various digitalization technologies, digitalization transformation has become the direction of enterprise transformation. Moreover, carrying out green innovation enables enterprises to contribute to sustainable development, protect public health, and fulfill social responsibility, which are the ardent expectations of today's internal and external stakeholders. Analyzing how enterprises practicing digitalization transformation can effectively promote green innovation performance is of great value to enterprise management and economic development. On the basis of the existing data, the enterprise digitalization is decomposed into 6 features from the perspective of production and operation, and then the above features are quantified according to the multi-source objective data from Chinese listed industrial enterprises. In order to explore the complex impact of enterprise digitalization on green innovation performance under multi-features, we also use the cluster analysis, the decision tree model, and other machine learning algorithms to mine the information from these research objects. Through the above work, we draw some interesting knowledge, some of which echo previous research, and some that can only be obtained through our research. The detailed conclusions are as follows:

(1) The more advanced digitalization transformation the enterprises have, the more possible for high green innovation performance to be achieved. This conclusion echoes the existing research, and deepens the study on the topic between digitalization and green innovation together from the micro perspective (31). From the analysis of clustering results, an enterprise with an advanced digitalization transformation process usually corresponds to a higher green innovation performance. In addition, the decision trees of different digitalization transformation situations indicate that enterprise digitalization features have a positive impact on the green innovation performance. For enterprises with an advanced digitalization transformation, their green innovation performance can be improved at a lower cost and through appropriate digitalization transformation ideas, and the corresponding digitalization features have a stronger positive impact on green innovation performance. With the deepening of the digitalization transformation process, enterprises can promote green innovation performance through mechanisms such as improving communication efficiency and enhancing knowledge integration capabilities to help them fulfill their social responsibilities (42).
(2) Digitalization innovation is the digitalization element with the strongest influenceability on green innovation performance. From heterogeneous decision tree analysis results, digitalization innovation output is the most important digitalization factor that causes enterprises to improve green innovation performance, which has the strongest promotion effect on enterprises in different digitalization transformation processes. Moreover, for enterprises with more advanced digitalization transformation, digitalization innovation input is another focus deserving attention. Both digitalization innovation and green innovation belong to the category of innovation in essence, so they require similar input resources and their outputs are of considerable value. Therefore, enterprises attaching importance to digitalization innovation will have a “ripple effect” on green innovation.
(3) With the advancement of digitalization transformation, enterprises should also focus on digitalization innovation input and digitalization operation output, otherwise they should focus on digitalization management and digitalization operation output. Compared with optimal distinctiveness thinking, enterprises with different digitalization situations have unique digitalization transformation paths that help improve green innovation performance. This research shows that, in addition to improving digitalization innovation output, ensuring sufficient digitalization operation output and digitalization innovation input are feasible ideas for enterprises that have excellent digitalization already to help improve green innovation performance. In contrast, enterprises with a relatively poor performance in digitalization are not advised to place too much emphasis on digitalization innovation investment, but should pay more attention to digitalization management, so as to start a top-down digitalization transformation to help improve green innovation performance. Therefore, with a clear understanding of their own digitalization transformation situation, enterprises should determine whether the focus should be digitalization innovation input or digitalization management, so as to help improve green innovation performance with their unique digitalization transformation process.

### 6.2. Management and policy enlightenments

The conclusions of this research can also provide the following implications for enterprise managers and policy makers about how enterprises should practice digitalization transformation to help improve green innovation performance.

Enterprise managers can learn from the digitalization deconstruction method proposed in this research to comprehensively and systematically understand their own digitalization transformation process and evaluate their own digitalization competitiveness. While pursuing green innovation performance, enterprises must actively improve their digitalization level, and they need to pay special attention to digitalization-related operating income and innovation output. For enterprises that have achieved certain achievements in digitalization, in addition to paying attention to the above two digitalization features, they should also strengthen the injection of digitalization-related innovation resources to achieve the “ripple effect” on their green innovation performance. Enterprises that lag behind in the process of digitalization transformation should turn their emphasis to digitalization management, make its “mindsponge” mechanism closer to digitalization, and help further the development of green innovation. Moreover, the pharmaceutical industry and the food industry are closely related to public health, so we combine our research with that of He et al. (26) and Thøgersen and Zhou (54) to make these recommendations. Enterprises belonging to the pharmaceutical industry should actively integrate digitalization and green concepts into production operations, and help enterprises to innovate activities more conducive to sustainable development. Enterprises in the food industry can consider selling organic products to consumers based on digitalization marketing technology and platforms, increasing the sales of “digitalization-green” products, and helping to achieve green innovation.

For policy makers, when promulgating laws and regulations related to environmental protection and green innovation, they should encourage enterprises to promote digitalization technology in production processes, management methods, innovation processes, and other aspects. Governments should also actively build a digitalization innovation environment and create a digitalization innovation atmosphere, so as to assist enterprises in green innovation and fulfilling their necessary social responsibilities. For enterprises that are undergoing a digitalization transformation and attach importance to green innovation, the government can provide policy support such as subsidies to help enterprises adjust their attitudes and management methods toward digitalization. Administrations can also use other economic means to help enterprises consume digitalization products and help improve the performance of green innovation in the supply chain. In addition, the office management department that provides digitalization-related support to enterprises should not be too hasty, but ought to take a longer period of time to investigate enterprises that actively inject digitalization-related resources.

### 6.3. Limitations and future research

This study provides new results for the topic of enterprise digitalization and green innovation through this research, but there are still the following areas for improvement. First, though digitalization transformation should be implemented by all enterprises, another focus of this study is green innovation performance. Therefore, we only discuss industrial enterprises, not those in the service industry. When other scholars in the future study digitalization-related topics, they can expand the research samples of other industries, and continue to explore the complex relationship between enterprise digitalization and other variables. Second, the conclusion has already mentioned that digitalization innovation input and output, as well as digitalization management and digitalization operation output, are of great significance to green innovation performance. However, due to space constraints and the focus of this study, the above features cannot be further subdivided. In the future, researchers can continue to decompose the above-mentioned digitalization features and explore their possible links with green innovation of enterprises. Third, we only lock the time span at one lag period between digitalization features and green innovation performance, but it is not discussed here due to method and space limitations. In the future, researchers can consider expanding the sample and introduce time factors as much as possible to deepen the related research.

## Data availability statement

The original contributions presented in the study are included in the article/supplementary material, further inquiries can be directed to the corresponding author.

## Author contributions

HL: conceptualization, methodology, design of the research, and supervision. HT: data curation, validation, design of the research, and writing the original draft. WZ: writing-review and editing and supervision. XW: writing-review and editing, validation, and supervision. All authors contributed to the article and approved the submitted version.

## Funding

This research has been supported by the National Natural Science Foundation of China (71771094), the Project of Natural Science Foundation of Fujian Province of China (2019J01067), and the Project of Social Science Planning of Fujian Province of China (FJ2020B088).

## Conflict of interest

The authors declare that the research was conducted in the absence of any commercial or financial relationships that could be construed as a potential conflict of interest.

## Publisher's note

All claims expressed in this article are solely those of the authors and do not necessarily represent those of their affiliated organizations, or those of the publisher, the editors and the reviewers. Any product that may be evaluated in this article, or claim that may be made by its manufacturer, is not guaranteed or endorsed by the publisher.
